# Evidence for extender oils in thermally generated tyre nanoparticles

**DOI:** 10.1039/d6em00472e

**Published:** 2026-08-25

**Authors:** Joshua Hassim, Siriel Saladin, Chiara Giorio, Adam Boies

**Affiliations:** a Department of Engineering, University of Cambridge UK aboies@stanford.edu; b Yusuf Hamied Department of Chemistry, University of Cambridge UK; c Department of Mechanical Engineering, Stanford University Stanford CA 94305 USA

## Abstract

Tyre-derived nanoparticles are an increasingly acknowledged component of road traffic emissions due to their potential health impacts. Although nanoparticle formation from tyres has frequently been attributed to evaporation–condensation processes, the chemical identity of the vapour-phase precursors remains unknown. In this work, we combined controlled thermal generation, solvent extraction, and nuclear magnetic resonance (NMR) spectroscopy to investigate thermally generated tyre nanoparticles. Cryomilled passenger car tyre tread was heated in a tube furnace, isolating thermal effects in the absence of mechanical abrasion. A clear onset of nanoparticle formation was observed above approximately 110 °C to 130 °C. Extracting the tyre tread with chloroform reduced cumulative nanoparticle emissions at 200 °C by 99% and 70% for two different tyre samples, indicating that nanoparticle formation at this temperature is dominated by solvent-accessible tyre constituents rather than the cross-linked rubber matrix. ^1^H and ^13^C NMR spectra of the generated nanoparticles were dominated by aliphatic hydrocarbon signals inconsistent with rubber polymer-derived molecules. Instead, the spectra matched the composition of common tyre extender oils, an essential component of passenger car tyre tread present at mass fractions on the order of 20%. Our results indicate that thermally generated tyre nanoparticles in the pre-pyrolysis regime originate predominantly from mobile, solvent-accessible hydrocarbon species consistent with extender oils. These findings provide a basis for risk assessments and toxicological evaluations of tyre wear nanoparticles, although microscopic temperatures at the tyre–road interface remain unknown and may extend beyond commonly assumed ranges.

## Abstract 2

Extraction and NMR analysis identify extender oils as a source of thermally generated tyre nanoparticles.

## Graphical abstract

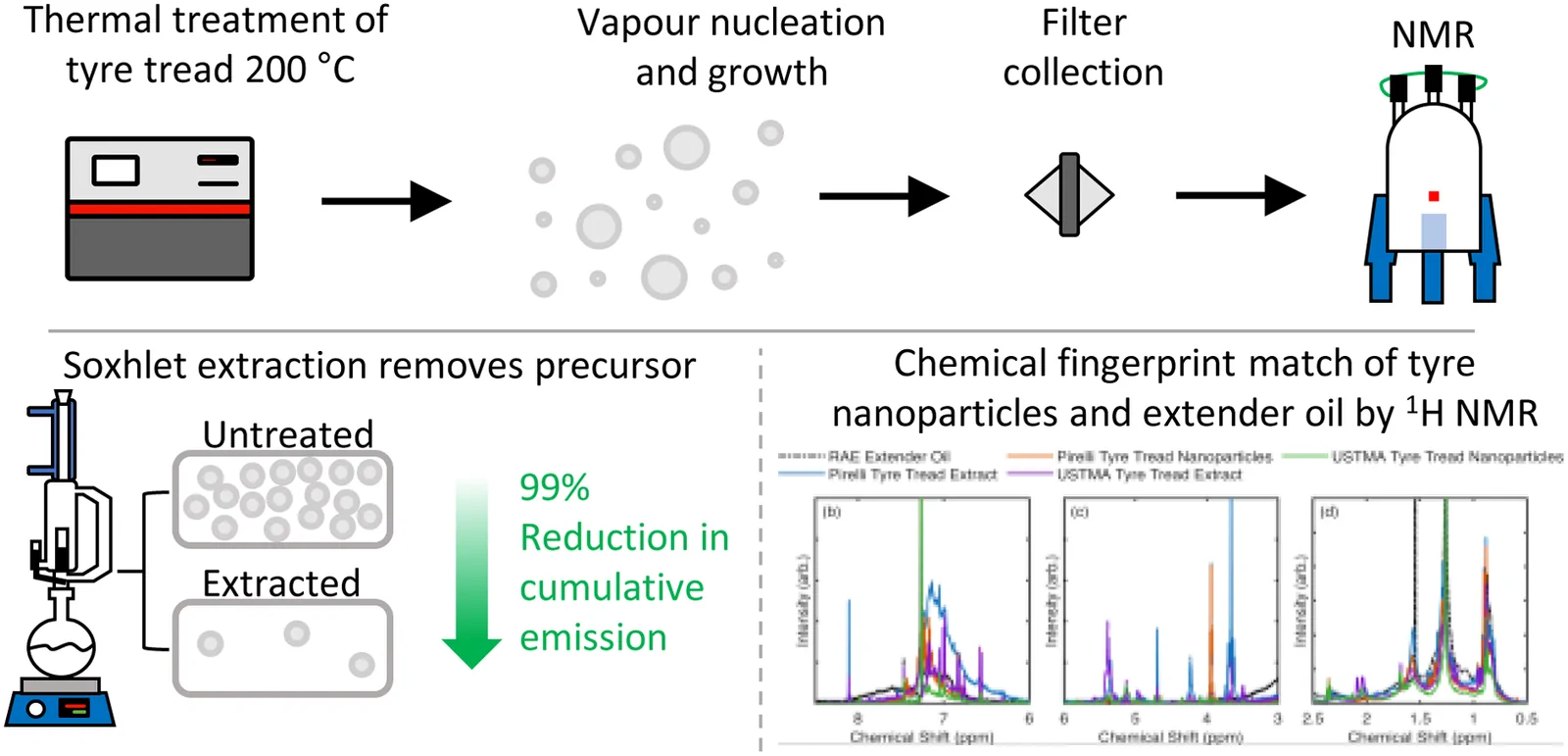

**Environmental significance**

Airborne tyre-derived nanoparticles are a recognised component of non-exhaust traffic emissions, but their composition and potential health impacts remain unclear. This study investigates nanoparticle formation from heated tyre tread at temperatures below thermal decomposition. The results show that particle formation is predominantly driven by volatilisation and subsequent condensation of extender oils rather than the rubber matrix. By identifying a mechanistically robust pathway for nanoparticle formation, these findings provide a foundation for improved emission characterisation and health-related studies of tyre-derived airborne nanoparticles.

## 1 Introduction

Tyre and road wear is increasingly recognised as a source of airborne particles from road traffic.^1^ While research has historically focused on mass-based metrics, recent work has highlighted the importance of the ultrafine (<100 nm) fraction due to potential health relevance.^2,3^ Current emission estimates for tyre-associated particle number emissions from passenger vehicles span approximately 10^9^–10^13^ particles per kilometre per vehicle,^3–7^ noting that these values represent total aerosol number including volatile and semi-volatile material. For context, current European vehicle exhaust particle number limits, which apply only to solid particles remaining after removal of volatile components, are set at 6 × 10^11^ particles per kilometre.^8^

In contrast to these quantitative estimates, there is less insight into the sources and composition of tyre-derived nanoparticles. This is particularly evident when compared to larger tyre and road wear particles in the micrometre size range, for which both composition and formation pathways are better characterised. These larger particles are produced predominantly through mechanical abrasion and consist of heterogeneous composites of elastomeric polymer, carbon black, mineral fillers, and, under real-world conditions, embedded environmental material.^9^ Tyre tread of passenger cars typically contains natural and synthetic rubber (40–50 wt%), fillers such as carbon black and silica (30–35 wt%), extender oils (15–20 wt%), vulcanisation agents (2–5 wt%), and other additives.^10,11^ As a result, wear particles collected in environmental and laboratory settings broadly reflect the composition of the parent tread, with additional contributions from external material incorporation.^9^

The formation of nanoparticles from the tyre–road interface introduces an additional level of complexity. If such particles were generated through mechanical fragmentation, they would be expected to retain the chemical signature of the bulk tyre or road. However, currently available evidence indicates that the majority of tyre and road wear nanoparticles are semi-volatile and likely organic in nature.^2,4–6,12,13^ These observations are inconsistent with a purely mechanical fragmentation mechanism and instead support an evaporation–condensation pathway involving release and subsequent nucleation of semi-volatile tyre and road constituents.

Despite this emerging consensus, it remains unclear which tyre or road constituents contribute to nanoparticle formation. This limits the development of test materials for toxicology and risk assessment. Among the components of passenger car tyre tread, extender oils are a plausible but unconfirmed source. Extender oils are processing fluids added during tyre manufacture to improve rubber processability by lowering viscosity and acting as volume extenders. They typically account for 15–20 wt% of tread mass for passenger car tyres.^11^ Their composition is predominantly long-chain, single-bonded hydrocarbons with reduced aromatic content, reflecting regulatory limits on polycyclic aromatic hydrocarbons under REACH.^14^ As mobile phases within the rubber matrix, they may be preferentially redistributed or released under thermal and mechanical stress, making them a strong candidate precursor for nanoparticle formation. This hypothesis was first proposed by Cadle and Williams^15^ in 1978, but direct experimental evidence remains missing.

In this work, we investigate the origin of tyre-derived nanoparticles using a tube furnace to isolate the role of heat in the absence of mechanical abrasion. Solvent extraction is used to assess the role of mobile organic constituents in nanoparticle formation. To determine particle composition, generated nanoparticles were collected and analysed by nuclear magnetic resonance (NMR) spectroscopy, together with extender oils and tyre extracts. This approach tests whether extender oils constitute a dominant source of the volatile material responsible for nanoparticle formation and provides insight into the chemistry of tyre-derived nanoparticles.

## 2 Methodology

### 2.1 Materials

Two different samples of tyre tread were investigated in this study. The first sample is tyre tread from a Pirelli Cinturato P7 C2 passenger car summer tyre (205/55 R16). It was cryogenically milled (Retsch, liquid nitrogen) for 10 min at 5 Hz and then 15 min at 20 Hz in a 50 mL stainless steel jar with one 25 mm stainless steel ball. The second sample is cryomilled tyre tread obtained from the U.S. Tire Manufacturers Association (USTMA). It consists of cryogenically milled tyre tread material derived from a blend of passenger car, light-duty, and heavy-duty vehicle tyres, described by USTMA as representative of the tyre market in the United States of America. For the rest of this work, the tyre tread derived from the Pirelli Cinturato will be referred to as “Pirelli Tyre Tread” and the USTMA mixture as “USTMA Tyre Tread”. In both cases, the type and quantity of extender oil are not declared by the manufacturers.

In addition to the tyre materials, three types of extender oils commonly used in tyre formulations were studied: mild extracted solvate (MES), treated distillate aromatic extract (TDAE), and residual aromatic extract (RAE). These oils consist predominantly of long-chain hydrocarbon species, with typical molecular masses on the order of 500 g mol^−1^, based on manufacturer-provided information.

### 2.2 Soxhlet extraction

**Fig. 1:**
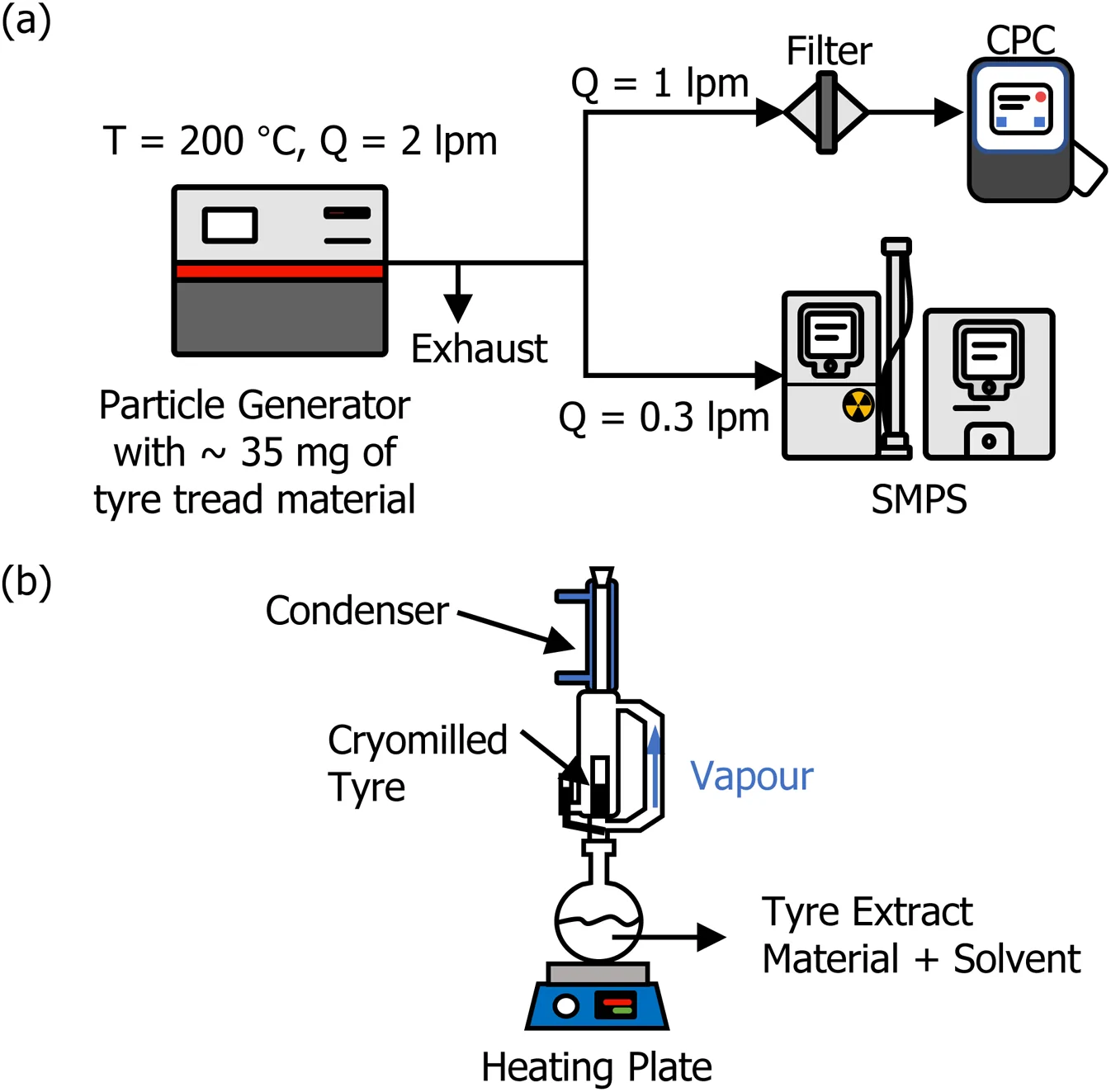
Experimental setup for tube-furnace experiments (a) and Soxhlet extraction (b). Flow rates along different sampling lines in (a) are denoted by *Q*. Aerosol instrumentation icons for this figure were obtained at https://github.com/tsipkens/aerosol-icon-project.

Both tyre tread samples were subjected to Soxhlet extraction using chloroform for 48 h (Fig. 1b). The non-extractable tyre material was dried at 60 °C for two days. The extract was yellow, and its solvent was removed at 60 °C, yielding a dark oil residue.

### 2.3 Tube furnace experiments

The tyre samples were heated in a vertical tube furnace (Fig. 1a) called the Silver Particle Generator (SPG) from Catalytic Instruments.^16^ The furnace was operated under a constant air flow of 2 l min^−1^ with no additional dilution flow.

Initial experiments were performed to investigate the onset of nanoparticle generation and the evolution of particle size distributions as a function of furnace temperature. For these experiments, approximately 100 mg of untreated cryomilled tyre tread material was loaded into the furnace tube. The furnace temperature was incrementally increased from 50 °C to 200 °C in 10 °C increments. After 5 minutes of thermal stabilisation at each temperature, three sequential 5 min Scanning Mobility Particle Sizer (SMPS) scans were recorded. Particle size distributions and integrated particle number concentrations were then averaged across the repeated scans at each temperature condition.

The remaining experiments utilised a smaller sample mass, with approximately 35 mg of cryomilled tyre tread material loaded into the furnace tube for each experiment. The reduced mass was selected to shorten particle emission decay times. Both untreated (non-extracted) and solvent-extracted tyre samples were analysed in the same way. The furnace was operated at a constant temperature of 200 °C. Before loading the sample into the SPG, the ceramic tubing was baked out at 400 °C until the particle number concentration emitted from the tube was zero and other impurities were absent. The furnace was ramped from room temperature to 200 °C over 10 min and subsequently held at 200 °C for a further 10 min to allow thermal stabilisation prior to commencing measurements. The aerosol stream exiting the furnace was subsequently cooled to approximately room temperature using a cooling coil positioned downstream of the furnace outlet. The aerosol-laden flow exiting the furnace was split downstream between (i) a filter (Sartorius, PTFE, 25 mm, 1.2 µm) for offline NMR chemical analysis and (ii) an SMPS for real-time particle size distribution measurements. The flow through the filter was controlled at 1 l min^−1^ using two condensation particle counters (CPC; Open CPC), confirming that particles were being adequately collected by the filter.

The SMPS system comprised a ^85^Kr neutraliser (TSI model 3077A), a differential mobility analyser (DMA; TSI model 3081, long column), and a condensation particle counter (CPC; TSI model 3752). The CPC was operated at a sample flow rate of 0.3 l min^−1^ to draw aerosol through the neutraliser and DMA. The DMA was operated with a sheath flow of 3 l min^−1^, corresponding to a mobility resolution of 10. Particle size distributions were measured over the mobility diameter range 13 nm to 750 nm, and total number concentrations were obtained by integrating the measured distributions over this size range.

### 2.4 NMR spectroscopy

The tyre nanoparticles collected on PTFE filters, the pristine extender oils, and the dry tyre extracts were dissolved in deuterated chloroform (CDCl_3_) at mass concentrations of approximately 5 mg mL^−1^.

^1^H and ^13^C NMR spectra were acquired on a Bruker Avance III HD 500 MHz spectrometer equipped with a dual ^13^C/^1^H helium-cooled cryoprobe. All measurements were performed at 298 K.

^1^H NMR spectra were recorded using a standard single-pulse sequence (zg30). Acquisition parameters included a 90° pulse width of 10.8 µs, a spectral width of 30 ppm (15 000 Hz), 8 scans, a relaxation delay of 1.0 s, and an acquisition time of 4.37 s.

^13^C NMR spectra were acquired using a power-gated decoupling sequence (zgpg30) to remove ^1^H–^13^C scalar couplings. The 90° pulse width was 10.5 µs, with 1024 scans and a relaxation delay of 2.0 s. The spectral width was 276 ppm (34 722 Hz), and the acquisition time was 3.02 s.

## 3 Results and discussion

### 3.1 Particle size distributions and emission onset

**Fig. 2:**
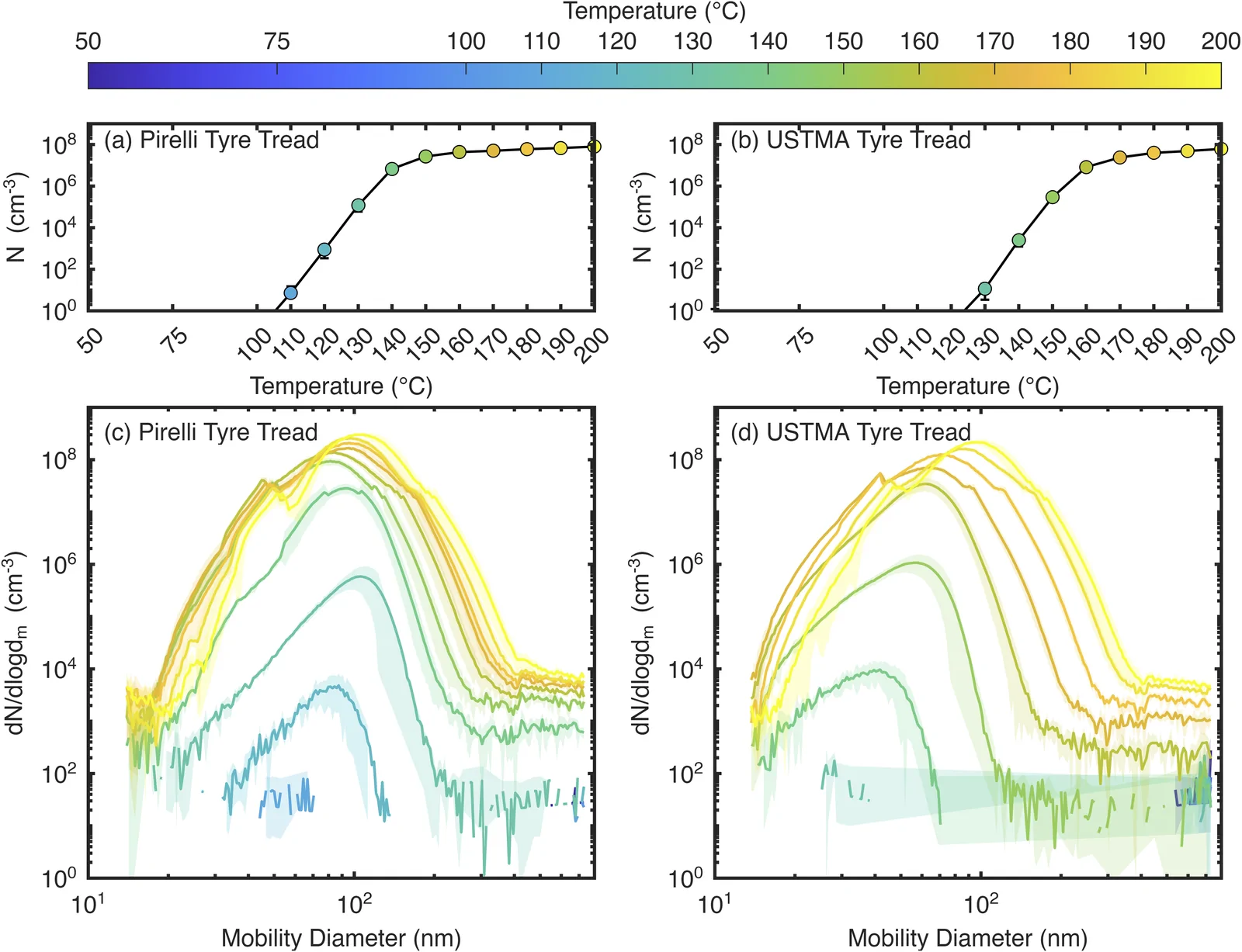
Temperature-resolved particle number concentrations and mobility size distributions measured using an SMPS for cryomilled tyre tread materials generated in a tube furnace. Panels (a) and (b) show the integrated particle number concentration (*N*) as a function of furnace temperature for Pirelli Tyre Tread and USTMA Tyre Tread, respectively. Panels (c) and (d) show the corresponding particle size distributions. Distributions are coloured according to furnace temperature, as indicated by the colour bar. Shaded regions represent ± 1*σ* variability between 3 repeat measurements at each furnace temperature.

Fig. 2 shows the integrated particle number concentration and the corresponding particle size distributions measured as a function of furnace temperature for two tyre tread materials: Pirelli Tyre Tread and USTMA Tyre Tread. The Pirelli Tyre Tread represents a commercially available passenger car tyre material, while the USTMA Tyre Tread is a blended material derived from multiple tyre types. Similar nanoparticle formation behaviour was observed for both materials, suggesting that the phenomenon is not specific to a single tyre formulation.

For both materials, negligible particle formation was observed at lower temperatures, followed by an increase in particle number concentration above an apparent onset temperature of approximately 110 °C to 130 °C. Here, the onset temperature should be interpreted only as an approximate indicator, since particle concentrations increased gradually rather than exhibiting a sharply defined threshold. This behaviour is broadly consistent with previous furnace-based studies of thermally generated tyre particles,^12,15^ although the precise onset temperature is expected to depend strongly on furnace-specific parameters including residence time, flow conditions, temperature gradients, and sample loading. Importantly, particle formation occurred in the absence of mechanical forces. This confirms that heat alone is sufficient to generate nanoparticles from tyre-derived materials, even at temperatures well below those typically associated with pyrolysis or other thermal degradation.^17^

Increasing furnace temperature led to a systematic increase in particle number concentration, accompanied by a substantial evolution of the particle size distribution. At lower temperatures, narrower distributions were observed, whereas at elevated temperatures the distributions became broader and shifted towards larger mobility diameters. Peak particle concentrations were typically observed in the approximate range of 70 nm to 120 nm, depending on both material type and furnace temperature.

It is important to emphasise that the measured particle size distributions are not intrinsic material properties, but instead reflect the specific generation pathway and surrounding thermodynamic conditions. In the present system, particle formation occurs *via* evaporation–condensation within a confined, low-dilution environment, generating high vapour concentrations that promote both nucleation and the subsequent growth of particles through condensation and coagulation. Consequently, the observed particle sizes are substantially larger than those typically reported in real-world measurements or tyre-in-drum studies,^2,6,13^ where dilution ratios, cooling rates, mixing conditions, and transport dynamics differ considerably. Parameters including vapour saturation ratio, residence time, and furnace temperature gradients all influence the resulting particle size distribution.

These experiments should therefore be interpreted as controlled demonstrations of the underlying nanoparticle formation mechanism rather than direct analogues of real-world tyre emissions. Specifically, the results demonstrate that (i) nanoparticle formation exhibits a distinct temperature threshold and (ii) thermal volatilisation followed by condensation can generate substantial concentrations of nanoscale particles from tyre tread without requiring the extreme temperatures typically associated with pyrolysis. While the absolute particle sizes differ from those observed in ambient or dynamometer studies, the qualitative behaviour provides important mechanistic insight into thermally driven nanoparticle formation processes.

Interpreting furnace-generated nanoparticles in the context of real-world tyre operation requires careful consideration. Laboratory studies generally report the onset of nanoparticle formation at temperatures above approximately 120 °C to 160 °C,^12,15^ whereas bulk tyre surface temperatures during conventional driving are typically below 50 °C, only approaching 80 °C to 100 °C under extreme operating conditions such as severe braking or sustained high lateral loading.^18,19^ Consequently, a direct equivalence between furnace temperatures and measured bulk tyre temperatures is not appropriate, particularly given the sustained temperature–time conditions used in furnace experiments.

However, this apparent discrepancy may be reconciled by considering highly localised transient thermal hotspots, or flash temperatures, generated at the tyre–road interface. Frictional dissipation and viscoelastic deformation at asperity-scale contacts can produce short-lived temperature spikes significantly exceeding the average measured tyre temperature.^20–22^

Comparing reported nanoparticle emission factors (∼1 × 10^11^ km per vehicle) with typical tyre wear mass losses (∼100 mg per km per vehicle)^23^ suggests that nanoparticle formation involves a mass fraction of about 0.00005–0.05% of the abraded tyre material (assuming 10 nm to 100 nm spherical particles). This implies that even if only an extremely small fraction of the friction interface experiences such temperatures, the observed magnitude of nanoparticle formation can still be accounted for.

In this work, furnace operation at temperatures up to 200 °C was therefore used as a controlled means of promoting volatilisation and increasing particle mass emission rates, enabling practical collection times while remaining below rubber pyrolysis temperatures.^24^ Importantly, any higher-temperature degradation processes (*e.g.* pyrolysis) necessarily pass through this intermediate thermal regime, supporting its relevance as a simplified model system for probing thermally driven nanoparticle generation mechanisms.

### 3.2 Solvent extraction

**Fig. 3:**
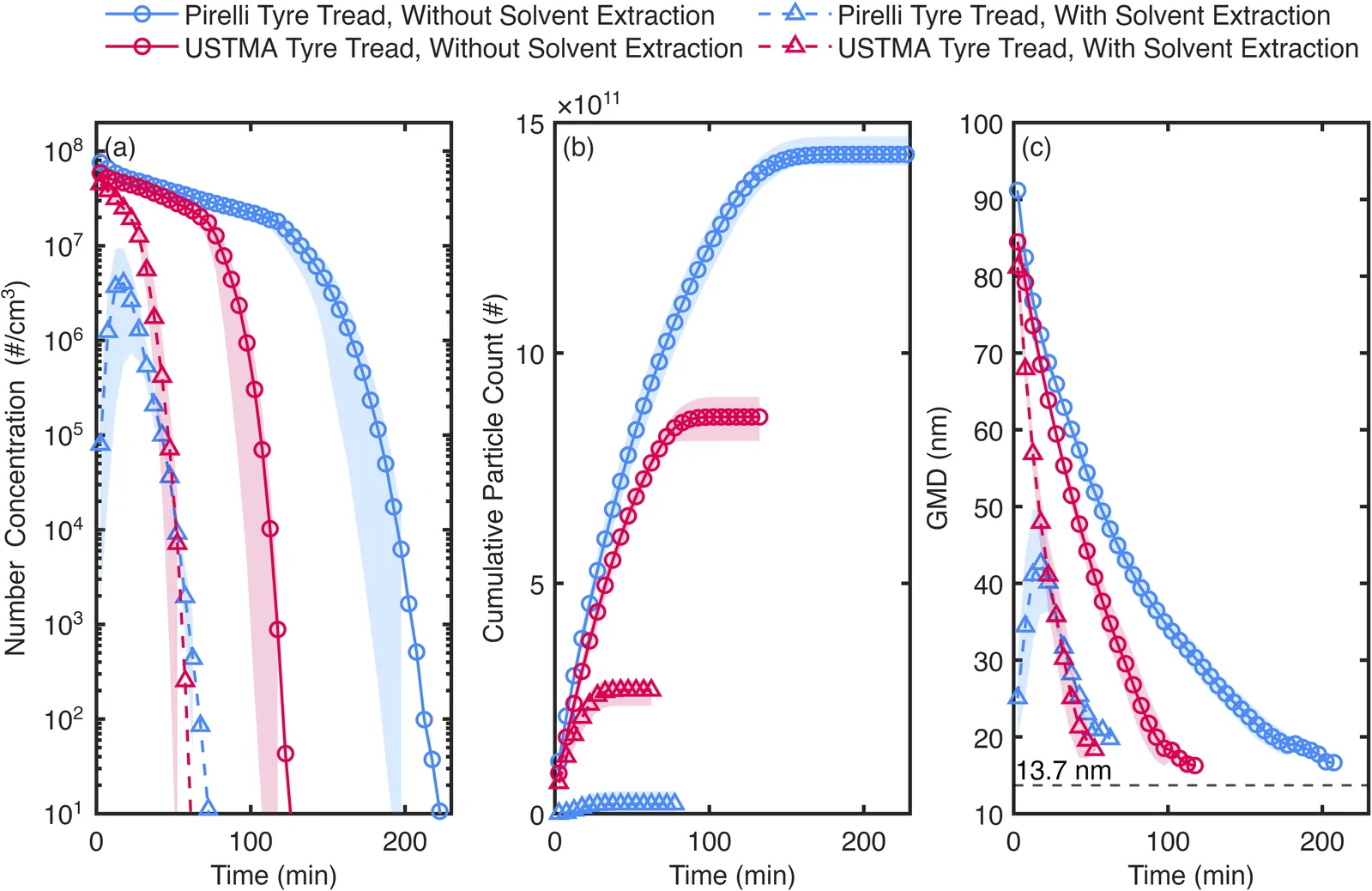
Temporal evolution of nanoparticle emissions from tyre tread samples at 200 °C. Both Pirelli Tyre Tread and USTMA Tyre Tread materials were tested with and without solvent extraction in chloroform. (a) Particle number concentration, calculated by integrating the measured SMPS size distribution. (b) Cumulative particle count emitted over the experiment. (c) Geometric mean diameter (GMD) of the emitted aerosol, calculated only when the particle number concentration exceeded 1000 cm^−3^. Indicated on figure (c) is the lowest particle size bin measured using the SMPS, 13.1 nm. Symbols and lines show the mean across three repeated experiments. Shaded regions indicate the variability between repeats at each time point (minimum-to-maximum range for particle number concentration and cumulative particle count; mean ± one standard deviation for GMD).

Solvent extraction was used to assess the role of solvent-accessible material in the thermally driven generation of nanoparticles from tyre tread. Fig. 3 compares the temporal evolution of particle number concentration, cumulative particle count, and geometric mean diameter for 35 mg of untreated and solvent-extracted Pirelli Tyre Tread and USTMA Tyre Tread samples heated at 200 °C.

The cumulative particle count (Fig. 3b) provides the clearest measure of the extraction effect. Solvent extraction reduced the total number of emitted particles by 99% for Pirelli Tyre Tread and 70% for USTMA Tyre Tread. Notably, Pirelli Tyre Tread lost approximately 26% and USTMA Tyre Tread 15% of the tyre tread mass during Soxhlet extraction. These mass losses are comparable to reported extender oil contents in passenger car tyre tread, which are typically in the range of 15–20 wt%.^11^ The substantially larger reduction in particle emissions observed for Pirelli Tyre Tread is consistent with its higher extracted mass fraction. Note that USTMA Tyre Tread also includes tyres from trucks and buses, which generally contain a higher fraction of natural rubber than passenger car tyres and may differ in extender oil content.^11^ The persistence of emissions after Soxhlet extraction indicates that aerosol-forming material remains within the tread matrix, suggesting either additional non-extractable contributions or incomplete extraction.

The GMD data further support this interpretation. Untreated samples produced particles with larger initial GMDs, followed by a progressive decrease in particle size as heating continued. This is consistent with a declining supply of condensable vapour: early in the experiment, higher vapour concentrations promote condensational growth, whereas later depletion limits growth and shifts the aerosol toward the lower measurable size range. Extracted samples generally produced smaller particles and approached the SMPS lower-bin limit more rapidly, consistent with reduced condensable vapour availability.

Overall, solvent extraction strongly suppresses thermally driven nanoparticle formation from tyre tread at 200 °C. Therefore, cross-linked rubber is unlikely to be a primary source of these emissions. Its network structure prevents quantitative solvent extraction, and the operating temperature of 200 °C is well below typical hydrocarbon pyrolysis thresholds,^24^ making extensive fragmentation of the polymer backbone improbable. In contrast, extender oils are more consistent with these observations, as they are mobile and present in passenger car tyre tread at mass fractions of 15–20 wt%.^11^

### 3.3 NMR spectroscopy

**Fig. 4:**
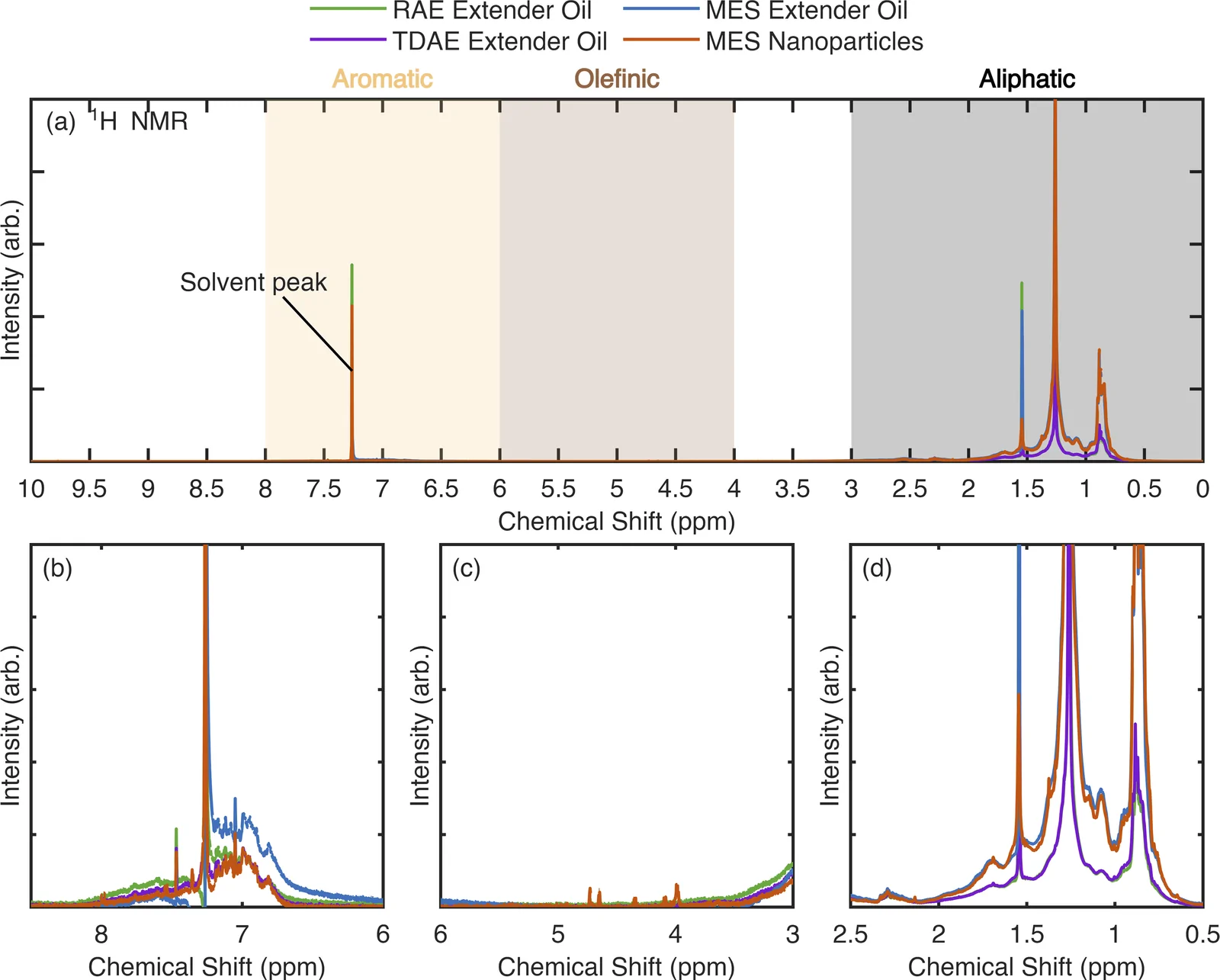
(a) ^1^H NMR spectra of three different pristine extender oils; MES, TDAE and RAE as well as nanoparticles from MES oil. Block-coloured regions indicate the aliphatic region (0–3 ppm), olefinic region (4–6 ppm), and aromatic region (6–8 ppm). (b–d) Enlarged sections of the ^1^H NMR spectra spanning 0.5–2.5, 3–6, and 6–8.5 ppm, respectively, to facilitate comparison of spectral features between samples.

Fig. 4 presents the ^1^H NMR spectra of three representative extender oils (MES, TDAE, and RAE), which are commonly used in tyre manufacture. In the ^1^H spectra (Fig. 4a), all samples exhibit dominant signal intensity in the aliphatic region (0–3 ppm), consistent with a high abundance of saturated hydrocarbon chains. Fig. 4d highlights the detailed structure of this region, where broad, overlapping resonances characteristic of methyl and methylene groups are observed, indicative of a complex mixture of linear and branched hydrocarbons. In contrast, only minor contributions are present in the olefinic (4–6 ppm, Fig. 4c) and aromatic (6–8 ppm, Fig. 4b) regions, demonstrating that unsaturated and aromatic species constitute a relatively small fraction of the overall composition.

The ^13^C NMR spectra (SI Fig. S1) provide complementary information on the underlying carbon framework. The spectra are dominated by signals in the 10–40 ppm range, corresponding to aliphatic (sp^3^) carbons. This pattern is characteristic of chemically complex hydrocarbon mixtures with varying chain lengths and degrees of branching. Notably, there is a negligible signal at higher chemical shifts, indicating the absence of significant aromatic, carbonyl, or oxygenated carbon functionalities. Together, the ^1^H and ^13^C data consistently demonstrate that all three extender oils are composed predominantly of saturated hydrocarbon structures, with only minor unsaturation. This is consistent with REACH regulations, which restrict the use of tyre extender oils with polycyclic aromatic hydrocarbons due to associated health risks.^14^

Additional experiments were conducted to test whether tyre extender oils alone can generate nanoparticles when heated in the same furnace. MES and RAE extender oils exhibited detectable nanoparticle emissions starting in the nanoparticle size range between 90 °C to 110 °C, not dissimilar to those of the tyre tread. Collected MES-derived nanoparticles showed NMR spectra (Fig. 4) almost indistinguishable from the bulk oil, indicating that the thermally generated nanoparticle material is chemically identical to the parent oil. This supports a volatilisation-driven mechanism in which pre-existing mobile hydrocarbons are released without chemical transformation.

The three extender oils exhibit similar ^1^H and ^13^C NMR spectra, with only minor differences, likely due to variations in chain length and branching. As their overall compositions are comparable, subsequent analysis uses a single representative oil (RAE) for comparison with tyre-derived materials.

**Fig. 5:**
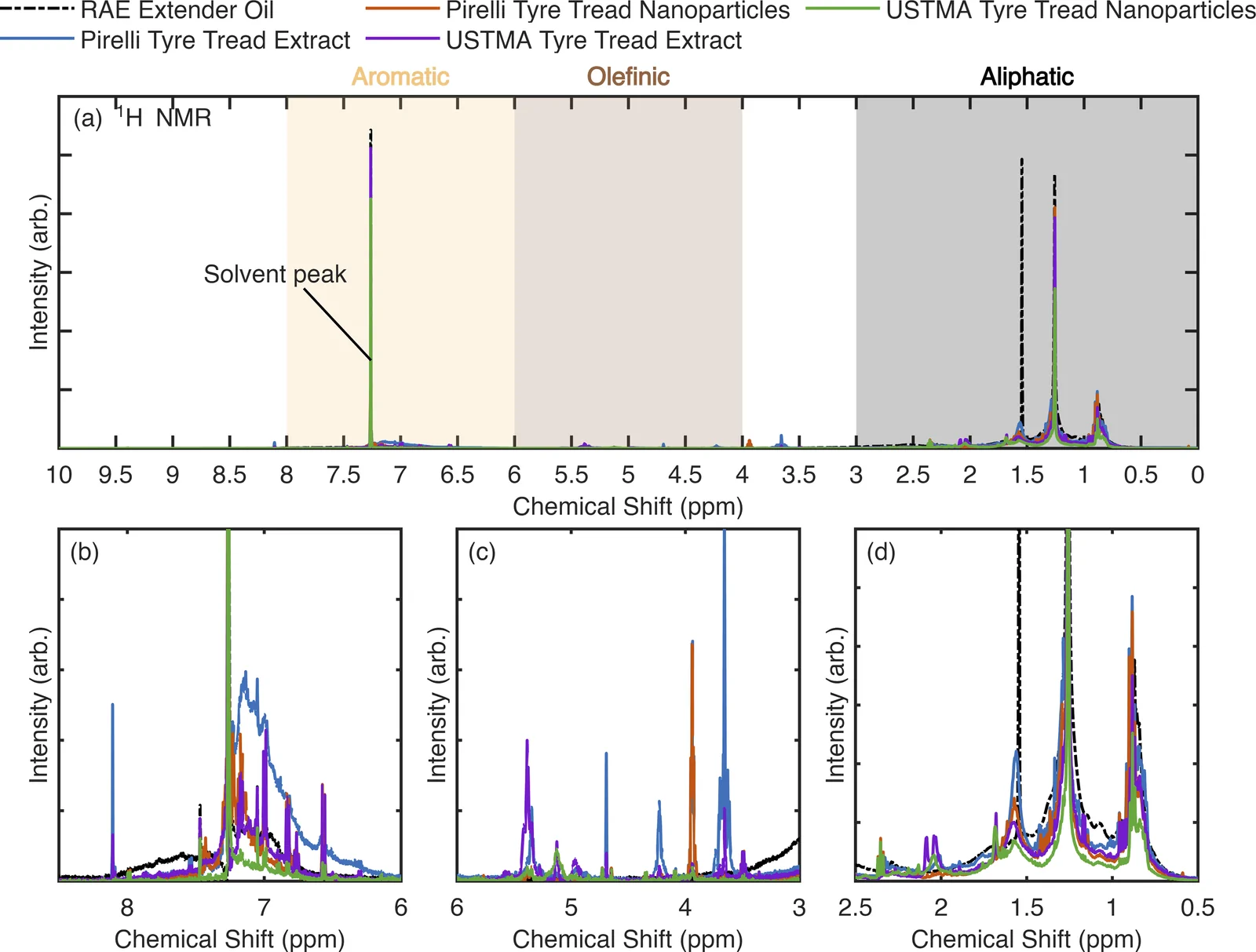
^1^H NMR spectra comparing RAE extender oil with tyre-derived materials from both Pirelli and USTMA tyre tread. Shown are spectra of extender oil (RAE), chloroform extracts of tyre tread, and tyre nanoparticles generated at 200 °C. (a) ^1^H NMR spectra with shaded regions indicating aliphatic (0–3 ppm), olefinic (4–6 ppm), and aromatic (6–8 ppm) chemical environments. (b–d) Enlarged sections of the ^1^H NMR spectra spanning 0.5–2.5, 3–6, and 6–8.5 ppm, respectively, to facilitate comparison of spectral features between samples.

Fig. 5 compares the ^1^H NMR spectra of RAE extender oil, chloroform extracts of Pirelli and USTMA tyre tread, and tyre nanoparticles generated at 200 °C from both tyre tread samples.

In the ^1^H spectra (Fig. 5a), all samples are dominated by signals in the aliphatic region (0–3 ppm, Fig. 5d), with minor contributions in the olefinic (4–6 ppm, Fig. 5c) and aromatic (6–8 ppm, Fig. 5b) regions. The results indicate that nanoparticles generated at 200 °C from both Pirelli and USTMA tyre treads are predominantly saturated hydrocarbon species, with only a minor contribution from unsaturated components. The tyre nanoparticle ^1^H spectra resemble those of their corresponding extracts and the RAE extender oil.

The ^13^C spectra (SI Fig. S2) highlight both similarities and differences between the samples. While all materials show strong and broadly comparable aliphatic signals (10–40 ppm), variations in the olefinic and aromatic regions (110–150 ppm) indicate compositional differences that are less clearly resolved in the corresponding ^1^H NMR spectra. Overall, the samples are chemically similar but not identical.

**Fig. 6:**
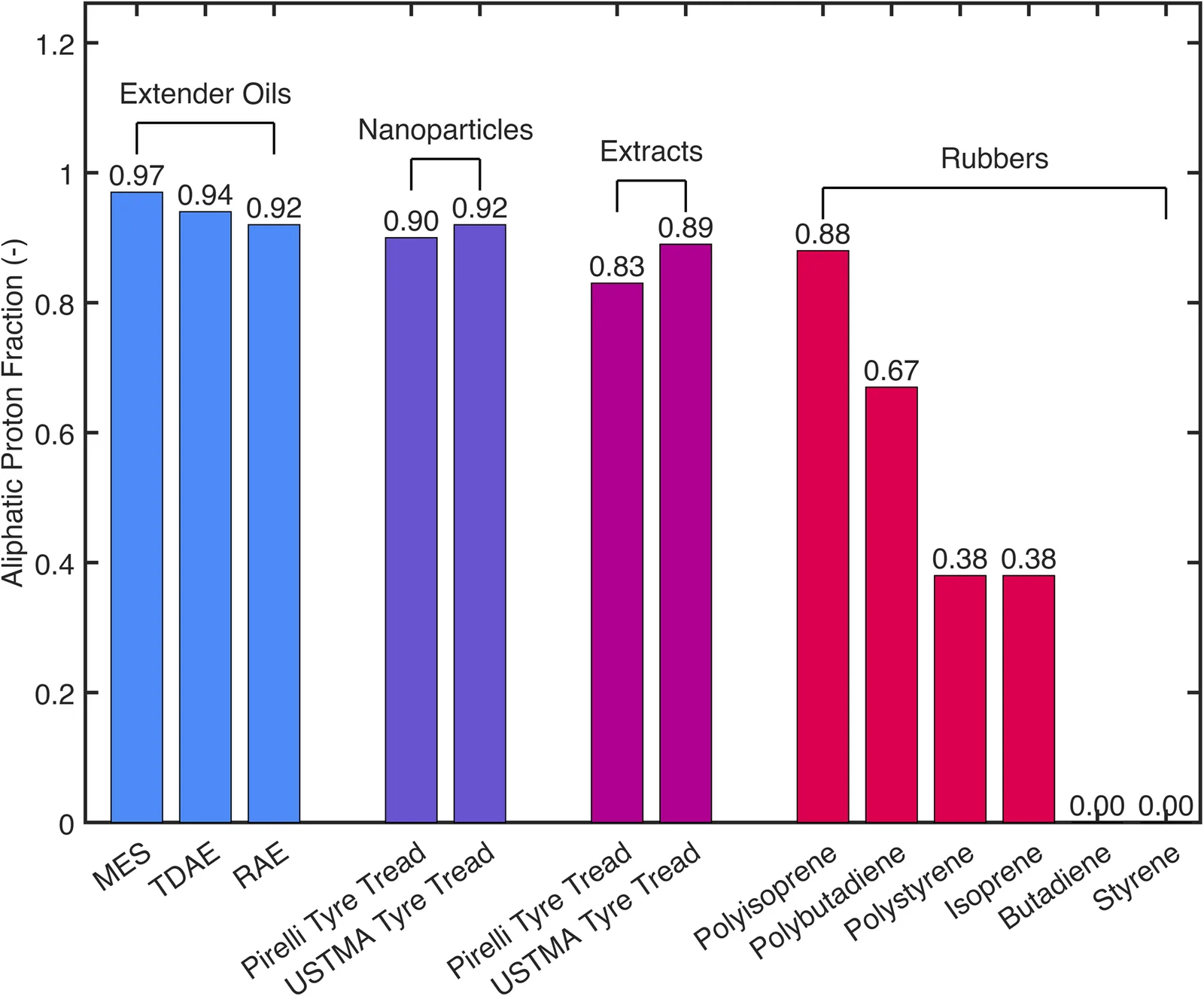
Comparison of aliphatic proton fractions for extender oils (MES, TDAE, and RAE), tyre-derived materials (nanoparticles and Soxhlet extracts from Pirelli Tyre Tread and USTMA Tyre Tread), and representative rubber polymers and monomers. For extender oils and tyre-derived samples, the aliphatic proton fraction was determined by integrating the ^1^H NMR spectra over the aliphatic region (0–3 ppm) and normalising by the total integrated proton signal (excluding solvent). In contrast, values for rubbers and monomers were calculated from their molecular structures by counting the numbers of aliphatic and total hydrogen atoms.

The aliphatic proton fractions (Fig. 6) of the extender oils (92–97%) are similar to the tyre nanoparticles from both materials (Pirelli Tyre Tread: 90%, USTMA Tyre Tread: 92%), whereas the corresponding tyre extracts exhibit slightly lower values (Pirelli Tyre Tread: 83%, USTMA Tyre Tread: 89%). In contrast, common rubber polymers (polyisoprene 88%, polybutadiene 67%, and polystyrene 38%), their monomers (isoprene 38%, butadiene 0%, and styrene 0%) and degradation products (4-vinylcyclohexene 58% and dipentene 81%) exhibit lower aliphatic fractions and, critically, stronger olefinic character that is not observed in the nanoparticle spectra. Although polyisoprene shows a relatively high aliphatic fraction, it contains significant olefinic content that is inconsistent with the measured spectra of the tyre nanoparticles.^25^

Notably, the aliphatic fraction is higher in the tyre nanoparticles than in the corresponding solvent extracts, and the NMR spectra show differences between the two fractions. Distinct ^1^H resonances shown in Fig. 5c are present in the tyre extract but are less pronounced in the nanoparticle spectra. This likely corresponds to more functionalised species that are extractable but participate to a lesser extent in the evaporation–condensation process, potentially due to insufficient or excessive volatility.

## 4 Research opportunities

The present study identifies extender oils as a precursor of thermally generated tyre nanoparticles under pre-pyrolysis conditions. This understanding opens avenues for future research.

Our results show that tyre nanoparticles generated at 200 °C are volatile and organic. Whether they remain volatile in ambient air or become less volatile through atmospheric ageing, for example, by reaction with ozone or UV radiation, remains unknown. Such ageing could transform initially liquid droplets into more persistent solid particles. These processes could be investigated under controlled laboratory conditions using atmospheric simulation chambers in combination with a thermal denuder or catalytic stripper.

Thermogravimetric analysis coupled with online chemical analysis further offers the opportunity to study temperature-resolved particle number and composition across the transition from pre-pyrolysis to pyrolysis.

It remains unclear whether real-world tyre nanoparticles are dominated by the evaporation of extender oils or by pyrolysis. Comparing tyres with different extender oil contents, for example, summer and winter tyres, could provide indirect evidence for the contribution of extender oils under real-world driving conditions. In our view, the extremely low nanoparticle masses observed under typical driving conditions make direct offline chemical characterisation using conventional techniques such as mass spectrometry, infrared spectroscopy, or NMR spectroscopy an impractical research direction.

The toxicity of tyre nanoparticles remains largely unknown. Future studies could investigate the biological effects of extender oils, while recognising that they represent only the extender oil-derived fraction of nanoparticle formation. Complementary studies could investigate the toxicity of pristine and aged thermally generated tyre nanoparticles produced under pre-pyrolysis and pyrolysis conditions. Future studies could also examine whether the nanoparticles persist in simulated lung fluids or dissolve after inhalation. If the nanoparticles dissolve, exposure would be governed by the low emitted mass of their molecular constituents rather than their high particle number.

## 5 Conclusions

We conclude that the tyre nanoparticles emitted in our furnace at 200 °C originate primarily from evaporation and condensation of extender oils rather than from rubber polymers, oligomers, monomers, or rubber degradation products. This conclusion is supported by (i) the strong reduction in particle formation after solvent extraction, consistent with removal of a solvent-accessible reservoir of nanoparticle precursors, (ii) the high mass fraction of extender oils in tyre tread, consistent with the observed mass losses during extraction, (iii) the ability of extender oil alone to form particles under the same thermal conditions, and (iv) the similarity between the NMR spectra of nanoparticles, extender oils, and tyre extracts, which differ clearly from rubber polymer-derived species.

Under real driving conditions, tyre wear involves a combination of mechanical and tribological processes at both the tyre and road surface. It remains unclear how our furnace-based findings translate to real-world tyre–road wear emissions. Nevertheless, they identify extender oil volatilisation as a mechanistically robust contributor to tyre nanoparticle formation. This is consistent with the early understanding of Cadle and Williams from 1978.^15^ Maximum real-world tyre temperatures may exceed 200 °C and could reach regimes where pyrolysis occurs, producing more unsaturated species with potentially different biological properties. However, the material would still pass through lower-temperature regimes where evaporation and condensation of extender oils contribute to nanoparticle formation.

## Author contributions

Joshua Hassim: conceptualisation, methodology, investigation, formal analysis, visualisation, and writing – original draft. Siriel Saladin: conceptualisation, methodology, investigation, formal analysis, visualisation and writing – original draft. Chiara Giorio: supervision, funding acquisition, resources, and writing – review and editing. Adam Boies: supervision, funding acquisition, and writing – review and editing.

## Conflicts of interest

There are no conflicts to declare.

## Supplementary Material

EM-028-D6EM00472E-s001

## Data Availability

Data supporting this article are available from the corresponding authors upon reasonable request. Supplementary information (SI) is available. See DOI: https://doi.org/10.1039/d6em00472e.
