# Supplementary material for: Evidence for extender oils in thermally generated tyre nanoparticles
Source: Environ Sci Process Impacts. 2026 Aug 25;28(10):2593–601. doi: 10.1039/d6em00472e (PMC13579560; doi:10.1039/d6em00472e)
Supplement: Supplementary file 1 [file EM-028-D6EM00472E-s001.pdf]

Supplementary Information (SI) for Environmental Science: Processes & Impacts.
This journal is © The Royal Society of Chemistry

Supporting Information: Evidence for extender oils in thermally generated tyre nanoparticles

Joshua Hassim[a,*], Siriel Saladin[b,*], Chiara Giorio[b], Adam Boies[a,c,**]

[a]*Department of Engineering, University of Cambridge, United Kingdom*
[b]*Yusuf Hamied Department of Chemistry, University of Cambridge*
[c]*Department of Mechanical Engineering, Stanford University, Stanford, CA 94305, USA*

---

*These authors contributed equally to this work.
**Corresponding author
Email address: aboies@stanford.edu (Adam Boies)

S1. Carbon NMR Spectra

S1.1. Extender Oil Carbon NMR

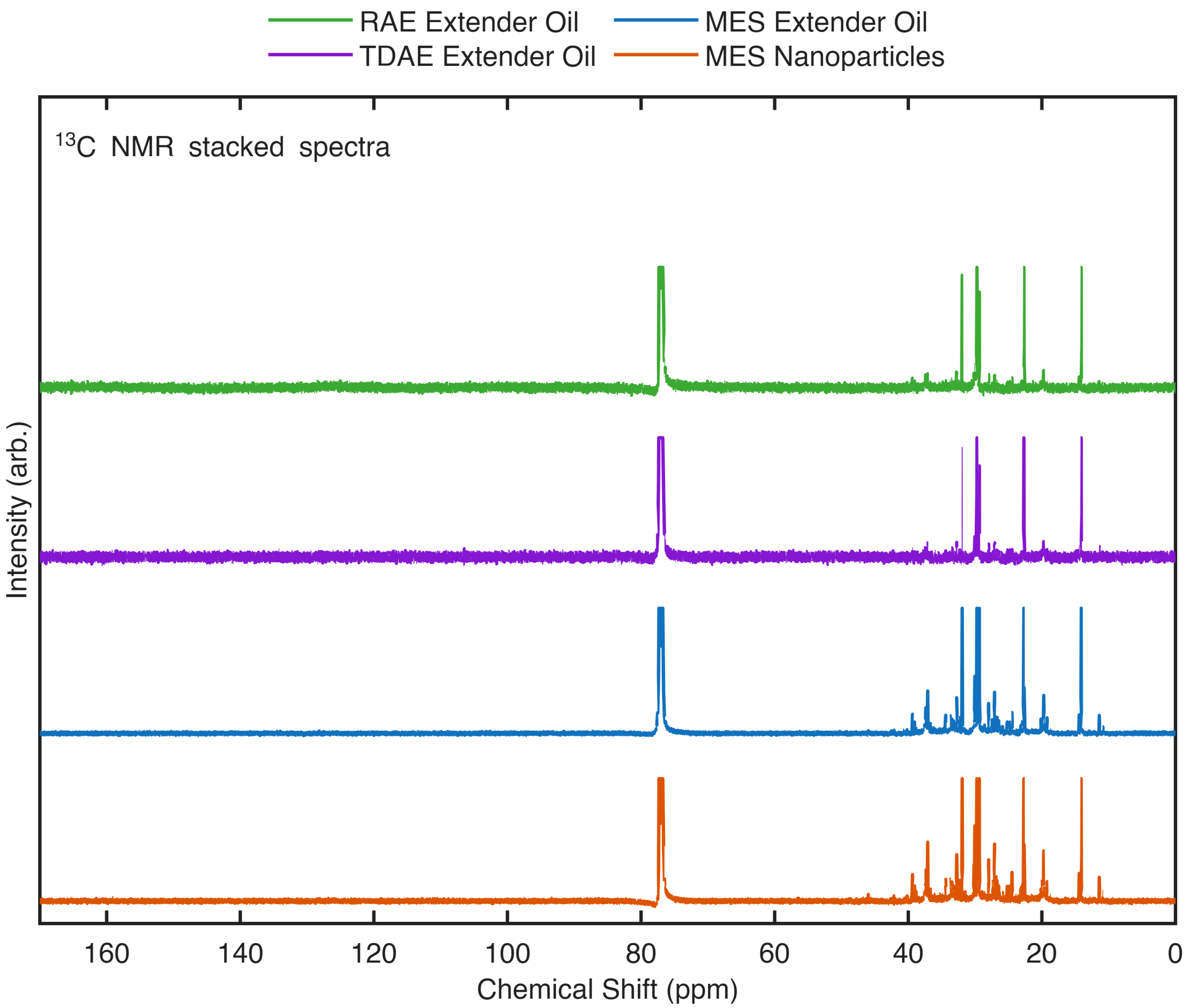

Figure S1: ^{13}C NMR spectra of the extender oils and MES nanoparticles. Spectra are vertically offset for clarity and coloured consistently with the corresponding ^{1}H NMR spectra shown in Figure 4. Solvent peak is present in all spectra at ~77 ppm.

S1.2. Nanoparticle and Extract Carbon NMR

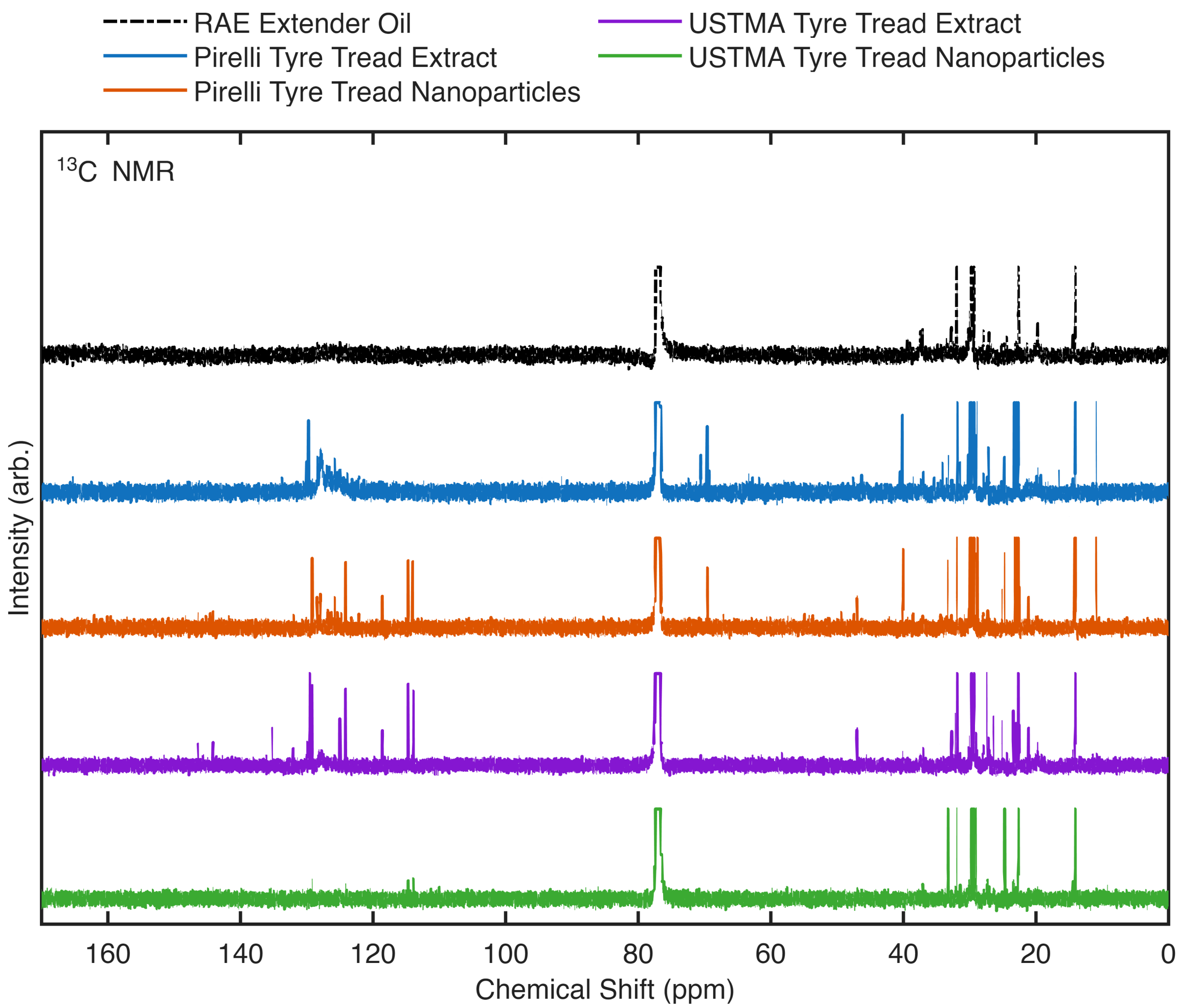

Figure S2: ^{13}C NMR spectra of the RAE extender oil, tyre nanoparticles and tyre extracts. Spectra are vertically offset for clarity and coloured consistently with the corresponding ^{1}H NMR spectra shown in Figure 5. Solvent peak is present in all spectra at ~77 ppm.
